# Strain-Specific Targeting and Destruction of Cells by Prions

**DOI:** 10.3390/biology13010057

**Published:** 2024-01-20

**Authors:** Sara M. Simmons, Jason C. Bartz

**Affiliations:** Department of Medical Microbiology and Immunology, School of Medicine, Creighton University, Omaha, NE 68178, USA; sarasimmons@creighton.edu

**Keywords:** prion disease, prion strain, prion neurodegeneration

## Abstract

**Simple Summary:**

Prions are novel infectious agents that consist only of protein. It is known that in the same host species, different strains of prion can occur that differ by the cells that are infected and killed. The mechanism responsible for these observations is only beginning to be understood. Two main ideas, which are not mutually exclusive, may provide a framework to understand strain targeting. The first possibility is that as prions spread throughout the CNS they reach strain-specific populations of cells that trigger cell death. The second mechanism is that prions exist in two distinct forms, a replicative form that switches to the production of a toxic form that results in the destruction of cells and the eventual onset of clinical signs of disease. Both neurons and glia may participate in both of these possibilities. The relative contributions of each mechanism have yet to be determined.

**Abstract:**

Prion diseases are caused by the disease-specific self-templating infectious conformation of the host-encoded prion protein, PrP^Sc^. Prion strains are operationally defined as a heritable phenotype of disease under controlled conditions. One of the hallmark phenotypes of prion strain diversity is tropism within and between tissues. A defining feature of prion strains is the regional distribution of PrP^Sc^ in the CNS. Additionally, in both natural and experimental prion disease, stark differences in the tropism of prions in secondary lymphoreticular system tissues occur. The mechanism underlying prion tropism is unknown; however, several possible hypotheses have been proposed. Clinical target areas are prion strain-specific populations of neurons within the CNS that are susceptible to neurodegeneration following the replication of prions past a toxic threshold. Alternatively, the switch from a replicative to toxic form of PrP^Sc^ may drive prion tropism. The normal form of the prion protein, PrP^C^, is required for prion formation. More recent evidence suggests that it can mediate prion and prion-like disease neurodegeneration. In vitro systems for prion formation have indicated that cellular cofactors contribute to prion formation. Since these cofactors can be strain specific, this has led to the hypothesis that the distribution of prion formation cofactors can influence prion tropism. Overall, there is evidence to support several mechanisms of prion strain tropism; however, a unified theory has yet to emerge.

## 1. Introduction

Prion diseases are inevitably fatal neurodegenerative diseases of animals that affect several species [1]. Prion diseases of animals include bovine spongiform encephalopathy (BSE or ‘mad cow’ disease), scrapie of sheep and goats, chronic wasting disease (CWD) that affects captive and free-ranging cervids, camel prion disease and transmissible mink encephalopathy (TME) of ranch-raised mink [2,3]. Prion diseases of humans include Gerstmann–Sträussler–Scheinker (GSS) disease, fatal familial insomnia (FFI), and Creutzfeldt–Jakob disease (CJD) [4]. Prion diseases are unique in biology in that they can occur in three etiologies. First, prions can exist as a sporadic disease with no known genetic or environmental basis. Second, prion disease can occur in familial forms that correspond to mutations in the host-encoded prion protein (PrP^C^) [5]. Third, under natural or experimental conditions, prions can be transmitted between individuals via numerous routes of infection including per os, inhalation and scarification of the dermis [6,7]. Prions can be zoonotic, as evidenced by the interspecies transmission of BSE to several species including Sapiens [8,9].

The transmissible agent of prion diseases is unique in biology and is composed of only protein [10,11]. The enrichment of prion infectivity led to the identification of a protein, PrP^Sc^, that was the major constituent of preparations that contained high levels of prion infectivity [12]. This protein, PrP^Sc^, is resistant to degradation by proteases and insoluble in detergents. Subsequent studies determined that PrP^Sc^ was encoded by the host protein, PrP^C^, eliminating a viral origin [13]. These observations led to the prion hypothesis that PrP^Sc^ is the infectious agent of prion diseases [14]. Subsequent studies reconstituting infectious prions from minimal non-infectious components confirmed the prion hypothesis [15,16,17,18,19,20]. Prion formation occurs when PrP^Sc^ encounters PrP^C^ and through an unknown mechanism can direct a global rearrangement of alpha helical structures present in PrP^C^ to parallel in-register intermolecular beta sheet structures in PrP^Sc^ [21,22,23,24]. The fragmentation of the growing PrP^Sc^ fibril provides new free ends of PrP^Sc^ to engage in subsequent rounds of binding and converting PrP^C^ to PrP^Sc^ [25]. This process can be recapitulated in vitro using protein misfolding cyclic amplification (PMCA), leading to the exponential propagation of PrP^Sc^ and prion infectivity [26,27].

Prion diseases exhibit strain diversity. Prion strains are operationally defined by heritable differences in the phenotype of disease under carefully controlled transmission conditions [28]. The phenotype of disease can include incubation period, clinical signs, distribution and intensity of neuropathology and tropism of PrP^Sc^. Tropism can include strain-specific differences in tissues infected, and within a given tissue, strain-specific differences in region or cell type where prions are detected [29,30,31,32]. While strains are best characterized in rodents, prion strain diversity has been observed in natural host species including Sapiens [33,34]. While initially difficult for the prion hypothesis to provide a mechanism to explain prion strain diversity [35,36], a wealth of indirect evidence supports the hypothesis that strain-specific differences in the conformation of PrP^Sc^ encode prion strain diversity [37,38,39,40,41,42]. Recent evidence using cryo electron microscopy has revealed that PrP^Sc^ from two distinct murine prion strains, which share the same primary amino acid sequence of PrP, have distinct structures of PrP^Sc^ [43,44]. This direct evidence provides the most compelling evidence to date that the conformation of PrP^Sc^ encodes for prion strain diversity. The relationship between the strain-specific conformations of PrP^Sc^ and the phenotype of disease is, however, poorly understood.

The neuropathology of prion infection includes spongiform degeneration, reactive gliosis, and neuronal death in the absence of a cellular inflammatory infiltrate. Spongiform degeneration is caused by the development and merging of interneuronal vacuoles that is observed in nearly all prion diseases and may be a consequence of the unfolded protein response dysregulation phosphoinositide kinase PIKfyve [45]. However, in some instances, spongiform degeneration is not a prominent feature of prion infection [46,47]. Prion diseases are characterized by the activation of both astroglia and microglia [48,49,50]. Astrocytes can support prion formation, and the role of astrocytes in neurodegeneration is becoming increasingly clear [51]. The restriction of PrP^C^ expression to astrocytes can result in prion formation in these cells; however, they do not undergo activation and neurodegeneration, and the development of clinical signs of prion infection is not observed [52]. These recent findings are consistent with previous work showing that brain grafts that express high levels of PrP^C^ in a PrP^−/−^ host undergo prion formation and the histological changes associated with prion infection, while PrP^Sc^ from the graft that entered the PrP^−/−^ brain failed to cause neurodegeneration or glial activation [53]. Importantly, the pattern of reactive astrocytes is strain specific in a large cohort of murine-adapted prion strains [54]. The incubation period of prion infection corresponds to the degree of astrocyte activation and the fact that inhibition of the unfolded protein response in astrocytes can prevent neuronal loss [55,56]. Overall, these observations suggest that PrP^C^ plays a critical role in neurodegeneration and that the interplay between neurons and glia is an important determinant in the outcome of disease.

## 2. Prion Strain Targeting

Neuropathological changes and PrP^Sc^ deposition patterns can vary based on prion strain, in part due to cellular targeting to a preferred neuronal subset. Different strains exhibit different cellular tropisms, evidenced by a multitude of studies. While many human prion strains cause a preferential and marked loss of PV+ neurons, fatal familial insomnia spares this subset within both the hippocampus and temporal cortex while also helping to mediate the loss within the frontal cortex [57]. PrP^Sc^ deposition patterns have been examined to differentiate between prion strains. Experiments on cultured organotypic cerebellar slices displayed strain-specific PrP^Sc^ deposition patterns [58]. In addition, patterns of PrP^Sc^ deposition from sheep infected with various prion strains have been used to reliably distinguish between various prion strains [59]. Genotype may also play a role in the degree of PrP^Sc^ deposition seen within these strain-specific patterns. Polymorphisms, associated with FFI, that confer longer disease duration tend to extend PrP^Sc^ deposition to areas of the brain outside of those affected in those with a shorter duration of disease [60]. Taken together, these experiments highlight the preferential cellular targeting of prion strains to certain areas within the brain, evidenced by strain-specific PrP^Sc^ deposition profiles.

Prion strain-specific differences in tissue tropism are observed. Prion conversion and deposition occurs in secondary lymphoreticular system tissues but has also been observed in other locations (e.g., skeletal muscle, fat) [61,62,63,64,65,66,67,68,69,70]. The extraneural distribution of PrP^Sc^ is not observed in all prion diseases and can differ based on prion strain. Sheep naturally infected with classic or Nor98 (atypical) scrapie have differences in the distribution of PrP^Sc^ in the spleen and lymph nodes [71,72]. A similar phenomenon is observed in hamsters infected with either the hyper (HY) or drowsy (DY) strains of hamster-adapted transmissible mink encephalopathy (TME) [37,73]. A widespread distribution of PrP^Sc^ is observed in HY TME-infected animals with the robust accumulation of PrP^Sc^ in the spleen, lymph nodes and skeletal muscle [62,74]. In contrast, prion infectivity and PrP^Sc^ is not detected in extraneural tissues in DY TME-infected animals [75]. This failure to establish infection is not due to a failure of prion transport as inoculum DY PrP^Sc^ can cross the epithelium and reach draining lymphatics following extranasal infection and the spleen following intraperitoneal infection [29]. Additionally, the spleen contains all the necessary components for DY TME formation as the spleen can support DY PrP^Sc^ formation in vitro during protein misfolding cyclic amplification [29]. Overall, strain-specific differences in prion tropism occur; however, the mechanism(s) are poorly described. The remainder of the review will explore potential mechanisms to explain prion tropism.

## 3. Clinical Target Areas

**Figure 1:**
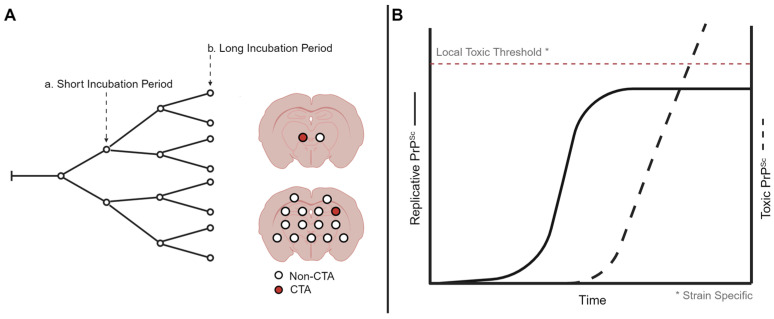
Models of prion strain-specific targeting and neurodegeneration. (**A**) Strain-specific distribution of prion neuropathology may be due to differences in prion spread when population of neurons are reached and destroyed. Here, a short incubation period strain reaches the clinical target areas earlier compared to a long incubation period strain, resulting in regional differences in neuropathology. (**B**) At the cellular level, prion formation may start with a replicative form that plateaus, resulting in the production of a toxic prion species. The level needed for the toxic species to result in cell death may be strain and cell type specific. Created with BioRender.com.

Clinical target areas (CTAs) are hypothesized to be areas of the brain that, when reached by PrP^Sc^, lead to the development of clinical signs and ultimately the death of the host [76]. The time between the onset of prion replication within the brain and the development of clinical signs is known as the replication phase. The targeting of prions to CTAs determines the length of the replication phase within the brain; CTAs constitute a minority of the neurons within the brain and are strain specific as prion strains can differ in the clinical presentation of disease (Figure 1A). Altering the route of inoculation altered the length of the replication phase, suggesting that the pathway to a proposed CTA may vary in complexity [76,77].

Prions spread along defined neuroanatomical pathways following various inoculation routes independent of prion strain. Neuroinvasion, following peripheral routes of inoculation (e.g., intraperitoneal, per os), establish infection in follicular dendritic cells in the spleen and visceral lymph nodes and continued along autonomic nerves before reaching the midthoracic spinal cord [66,67,76,78,79,80,81,82,83,84,85,86]. Intralingual muscle inoculation provided the first evidence for retrograde axonal transport independent of prion strain, with spread retrograde axonal transport via the hypoglossal nerve to the hypoglossal nucleus and subsequent transsynaptic transport to the nucleus of the solitary tract [87]. Building upon these findings, the direct inoculation of the sciatic nerve with three different hamster-adapted prion strains indicated they all used retrograde axonal transport along the same four descending motor tracts: reticulospinal, vestibulospinal, rubrospinal and corticospinal tracts [88,89]. These observations suggest that prion strain targeting CTAs is not due to differences in prion transport in the CNS. The strain-specific differences in neuropathology and PrP^Sc^ deposition may be due to differences in the progression of the spread of PrP^Sc^ due to strain-specific differences when the CTAs are reached. Strain-independent retrograde axonal transport is also observed with other non-PrP^Sc^ prion-like diseases such as Parkinson’s disease and amyotrophic lateral sclerosis. Murine wild-type α synuclein (α Syn) fibrils and human E46K α Syn fibrils, associated with Parkinson’s disease, both underwent retrograde axonal transport along vestibulospinal and rubrospinal descending motor tracts following sciatic nerve inoculation [90]. The sciatic nerve inoculation of mutant SOD1 brain homogenates into transgenic mice expressing G58-SOD1:YFP resulted in the retrograde axonal transport of pathology along three descending motor tracts: the reticulospinal, vestibulospinal and rubrospinal tracts [91].

Differences in the duration of the asymptomatic replication phase of prions in the CNS are influenced by the route of infection. The prion replication phase in the CNS following intraperitoneal (i.p.) inoculation is shorter compared to the replication phase following intracerebral (i.c.) inoculation [92]. The shorter replication phase observed following i.p. inoculation suggests a more direct pathway to CTAs from the midthoracic cord compared to the pathways following i.c. inoculation. Consistent with this observation, the intraspinal inoculation of mice with 139A prions showed a shorter replication phase compared to i.c. inoculation, indicating a potential difference in the complexity of the neuronal pathway to the CTA available with a given inoculation site [77]. While the targeting and formation of PrP^Sc^ that results in neurodegeneration within the CTA determines the onset of clinical signs, prion spread to non-CTA regions is hypothesized to contribute to prion infectivity titers and the distribution of spongiform degeneration [76]. As spongiform degeneration precedes the onsets of clinical signs of disease, this means that either spongiform degeneration per se does not lead to clinical signs, or that spongiform degeneration in combination with another neurodegenerative event precipitates the onset of clinical signs of disease. Since it is hypothesized that the clinical signs associated with prion disease are a result of the replication and pathology only found within the CTAs, the route of infection thus may alter the pathways available to CTAs after neuroinvasion and therefore the duration of the replicative phase of disease.

## 4. Replicative vs. Toxic forms of PrP^Sc^

Two distinct forms of PrP^Sc^, a replicative form and a toxic form, are hypothesized to determine the onset of clinical signs of prion disease. The uncoupled replication and toxicity hypothesis describes two distinct phases of prion pathogenesis [93]. The first phase is characterized by the exponential accumulation of PrP^Sc^ that eventually plateaus. The plateau in PrP^Sc^ abundance is reached prior to the onset of clinical signs of prion infection. The second phase confers toxicity that is mediated by a toxic form of PrP, termed PrP^L^. The toxic effects associated with this form of PrP are thought to occur only after surpassing a toxic threshold (Figure 1B). Thus, the initial rapid propagation of replicative PrP^Sc^ is not responsible for toxicity, and toxicity occurs after the formation of PrP^L^ to necessary threshold saturations.

The formation of PrP^L^ is hypothesized to be the result of off-target or intermediate forms of PrP^Sc^. Compared to PrP^Sc^, PrP^L^ may have reduced self-templating activity, therefore requiring an increased time to accumulate to toxic levels. This hypothesis predicts that the rate of PrP^L^ formation is influenced by the rate of PrP^Sc^ conversion and maturation [94]. With the increased conversion of PrP^Sc^, more intermediates are generated, including PrP^L^. Fluctuations in isoforms occur in two phases, with synaptic alterations and neuropathological changes occurring after an increase in PK-sensitive isoforms generated in phase 2 [95]. The hypothesis of defined toxic thresholds is bolstered by the findings that similar levels of PrP^Sc^ must be reached for clinical disease to progress in phase 2, regardless of PrP expression [95]. PrP^L^ may have a different conformation from PrP^Sc^ as both enriched PrP^Sc^ preparations and the sarkosyl treatment of RML-infected brain homogenates eliminated toxicity while maintaining prion infectivity. Collectively, these results are consistent with a toxic and replicative form of PrP^Sc^.

Observations in animals during the subclinical phase of disease may provide support for the uncoupled replication and toxic forms of PrP^Sc^ hypothesis. Animals with subclinical prion disease replicate PrP^Sc^ and can live a normal life span in the absence of clinical signs, including ataxia [96]. Subclinical prion disease occurs in mice inoculated with the hamster strain Sc237 as these mice were able to replicate PrP^Sc^ to high levels but failed to exhibit clinical signs of disease [97]. In addition, inoculation with low-dose inoculums of mouse strains into mice overexpressing PrP^C^ induced a subclinical disease state. Interestingly, in this study comparison of terminally ill high-titer inoculated and subclinical low-titer inoculated mice displayed similar levels of PrP^Sc^ within the brain stem [98]. If the rate of formation of PrP^L^ is decreased in subclinical disease due to species barrier effect or slowed replication, a small amount of PrP^L^ formed may undergo sufficient maturation into PrP^Sc^, thus preventing clinical disease by eliminating the source of toxicity. In both instances, the propagation of PrP^Sc^ alone was not able to induce clinical symptoms, suggesting that another toxic mechanism is required to elicit irreversible clinical signs and terminal disease.

Factors, in addition to PrP^L^, may be involved in the development of clinical prion disease. A prediction of the replicative and toxic PrP hypothesis is that the initial sites of neuroinvasion would be the first areas of the CNS to reach a plateau in infectivity, followed by the accumulation of PrP^L^ resulting in the onset of clinical signs of disease. This pattern, however, is not observed following extraneural routes of inoculation, where the initial site of neuroinvasion does not correspond with the development of clinical signs of disease [99]. For example, the inoculation of the sciatic nerve with HY TME prions results in the detection of PrP^Sc^ in ventral motor neurons (VMNs) in the lumbar spinal cord ipsilateral to the side of prion inoculation within 2 weeks postinfection [87,100]. From VMNs, PrP^Sc^ spreads along known neuroanatomical pathways until the onset of clinical signs 8 weeks later. The replicative vs. toxic PrP hypothesis would predict that hind limb motor deficits ipsilateral to the side of prion inoculation would be the first clinical sign of disease as VMNs are the first cell type infected and would first produce PrP^L^. What is observed, however, is clinical signs of hyperexcitability and cerebellar ataxia that is indistinguishable from other routes of infection [87,100]. A similar relationship between the initial deposition of PrP^Sc^ in VMNs and clinical disease is observed following the sciatic nerve inoculation of DY TME with animals developing clinical signs of progressive lethargy and not hind limb motor deficits. These observations constrain the properties of PrP^L^. First, as hind limb motor deficits are not observed, either PrP^L^ is not produced in VMNs or PrP^L^ is not toxic for VMNs. Second, as HY- and DY TME-infected animals have distinct clinical signs that occur independent of the route of infection, strain-specific forms of PrP^L^ that affect different populations of neurons are required [7,62,75,101]. Additionally, synaptotoxic forms of PrP^Sc^, presumably PrP^L^, are observed early during the pathogenesis of disease, prior to the plateauing of infectivity titers [102]. Overall, these observations suggest that host factors, in addition to PrP^L^ reaching local toxic thresholds, contribute to prion disease development.

## 5. Role of PrP^C^ in Neurotoxicity

PrP^C^ is a cell surface protein that is anchored to the cell membrane by a glycosylphosphatidylinositol (GPI) anchor [13,103,104]. During prion formation, conversion occurs as PrP^Sc^ uses available PrP^C^ as a substrate [22]. PrP^Sc^ serves as a template to misfold PrP^C^ into the pathogenic and misfolded form, PrP^Sc^. Two forms of PrP^C^ exist, C1 and C2, resulting from the proteolytic cleavage of PrP^C^. C1 is the soluble, C terminal fragment of PrP^C^ which has undergone α cleavage between amino acid residues 111/112, which eliminates the amyloidogenic region of residues 106–126 [105,106]. The C1 fragment is present in the brains of both healthy and prion-infected animals. While mice expressing C1 alone inoculated with RML failed to develop prion disease, mice co-expressing C1 and WT PrP^C^ resulted in a prolonged incubation period and the slower accumulation of PrP^Sc^ [107]. These experiments provided evidence for a potential protective role of the C1 fragment, as it cannot be used as a substrate for PrP^Sc^ formation. In contrast, C2 is the insoluble form of PrP that is associated with β cleavage. C2 retains the PrP residues associated with the amyloidogenic region and is found only in the brains of prion-infected animals [105,106]. Therefore, while α cleavage may play a protective role in prion disease, β cleavage is associated with either PrP^Sc^ or PrP^C^ and potentially contributes to disease.

The expression of PrP^C^ is required for prion propagation. This is evidenced by both Prnp^0/0^ and Prnp^0/+^ mice inoculated with mouse adapted scrapie. While PrP^−/−^ mice fail to develop prion disease and pathology, PrP^−/+^ mice have an increased incubation period when compared to WT mice [108]. Importantly, in PrP^−/+^ mice, an extended period of a prion infectivity plateau is observed, suggesting that PrP^C^ plays a role in the onset of neurodegeneration and clinical signs of disease [108]. Conversely, the overexpression of PrP^C^ shortens the incubation period of prion disease; however, it is unclear if this is due to an increased tempo of prion conversion or increased susceptibility to neurodegeneration [109,110,111,112].

Alterations in PrP^C^ levels occur during prion infection. A downregulation in PrP^C^ occurs prior to a plateau in infectivity in WT mice inoculated with RML prions [113,114]. This decrease in available substrate for conversion is hypothesized to result in a decrease in the rate of PrP^Sc^ formation. The plateau effect observed in PrP^Sc^ abundance was also observed in PMCA experiments, where it was shown that a higher quantity of PrP^C^ and lower quantity of PrP^Sc^ leads to an increase in the replication rate of PrP^Sc^, while the inverse is true when a higher quantity of PrP^Sc^ and lower quantity of PrP^C^ is used [114]. Overall, alterations in the abundance of PrP^C^ associated with prion disease may drive the plateau of infectivity seen in this proposed first phase of prion pathogenesis.

The expression of PrP^C^ is required for the development of prion neuropathology. To investigate the role of PrP^C^ expression and neurotoxicity, neural explants overexpressing PrP^C^ were engrafted into the brains of PrP^−/−^ mice. The intracerebral inoculation of these mice with RML prions did not result in the development of clinical signs of prion infection. It did, however, result in the development of prion formation and associated neuropathology in the graft expressing PrP^C^ and not in the PrP^−/−^ brain regions. Importantly, PrP^Sc^ produced in the engrafted brain migrated into the PrP^−/−^ regions but did not result in observable neuropathological changes [53]. As neuropathology is observed in the engrafted PrP^C^-expressing brain regions, it should contain both the replicative vs. toxic forms of PrP^Sc^; therefore, both forms should migrate into the PrP^−/−^ brain region. However, neuropathological changes are absent in the PrP^−/−^ brain region, leading to the inference that if PrP^L^ is escaping from the explant, it is devoid of its toxic properties in the absence of PrP^C^. This experiment highlights the necessity of PrP^C^ in neurodegeneration and its potential role as a mediator of neurotoxicity.

Regions within PrP^C^ may play a role in mediating prion neurodegeneration. The central region (CR) of PrP has been identified as a crucial region in the maintenance of the neuroprotective function of PrP^C^. In the absence of CR, residues 105–125, neurodegenerative phenotypes, are present in transgenic mice [115]. Deletions of the CR induce spontaneous ionic currents [116,117]. Experimentation with anti-PrP antibodies directed specifically towards the globular domain of PrP^C^ found the majority to cause neurotoxicity in COCS. One such antibody, POM1, causes neuronal loss in vitro and in vivo [117]. ICSM18, a globular domain antibody that binds to an epitope of PrP^C^ that overlaps the epitope of POM1, was also found to induce neurotoxicity in vivo [118]. The flexible N terminal region is hypothesized to play a role in the mediation of toxic effects associated with prion disease [117]. In experiments involving the comparison of hippocampal neuronal culture systems expressing PrP^C^ with deletions in the N terminal region (Δ23–31) and WT PrP^C^, the toxic effects associated with PrP^Sc^ application were eliminated in the absence of this N terminal region [119]. Antibodies to the N terminal region, POM2, can neutralize the toxic effects associated with both RML prion disease as well as globular domain ligands in cerebellar organotypic culture slices [120]. The role of the N terminal region in toxicity is highlighted by cells expressing PrP(N)-EGFP-GPI, where spontaneous ionic currents are induced [121]. These experiments illustrate the interplay between key regions of PrP^C^ that function in either neuroprotective or neurodegenerative capacities and the influence of PrP^C^ and PrP^Sc^ interactions on these functions.

PrP^C^ can act as a toxic mediator in prion-like diseases. PrP^C^ functions as one of the receptors for oligomeric amyloid β, Aβo, which is associated with Alzheimer’s disease [122]. The binding of Aβo to PrP^C^ initiates a toxic signaling pathway that culminates in neurodegeneration. The inhibition of long-term potentiation is blocked in both Prnp^0/0^ hippocampal slices treated with synthetic Aβo, as well as WT hippocampal slices pretreated with anti-PrP antibodies [122]. In vivo studies of Alzheimer’s disease transgenic mice revealed that while Aβ can still accumulate in mice lacking PrP^C^, axonal degeneration as well as impairments in memory and spatial learning are not observed [123]. The activation of Fyn, a Src family kinase, by the binding of Aβ to PrP^C^ initiates a signaling pathway resulting in the synaptic dysfunction associated with NMDA receptors. With a specificity for Aβo, the binding of Aβo to PrP^C^ activates Fyn, which in turn leads to an initial phosphorylation of a subunit of NMDA receptors, NR2B, which increases the amount of NMDA on the cell surface, ultimately leading to excitotoxicity [124]. This is not observed in neurons lacking the expression of PrP^C^, Fyn or in neurons treated with anti-PrP antibodies prior to Aβo exposure. Thus, this evidence collectively provides evidence for the necessity of PrP^C^ expression in mediating the toxic effects of Aβo binding.

## 6. Cellular Cofactors and Prion Formation

The formation of PrP^Sc^ is aided by cellular co-factors. Prion formation is recapitulated by PMCA, leading to the production of infectious prions that maintain the strain properties of the initial input prion strain [27,125,126,127]. This system has been used to investigate the requirements for prion formation and has found that phosphatidylethanolamine (PE) and RNA facilitate prion formation in vitro. PMCA conversion of three prion strains that contained PE as the cofactor resulted in the three strains converging into a single strain that was indistinguishable from them and that also differed from the parental strain [128]. Since the information for prion strain diversity is encoded in the conformation of PrP^Sc^, the cellular co-factors have been hypothesized to play a role in tropism. This system had identified strain-specific requirements in cellular co-factors for efficient in vitro formation. For example, differences in the effect of RNA in the formation of PrP^Sc^ correspond with prion strain, suggesting that cellular cofactors can influence the rate of prion formation [129,130,131]. As the rate of prion formation must exceed prion clearance, the distribution of cellular co-factors may influence prion tropism [29]. The role of strain-specific cofactors in PrP^Sc^ tropism in neurons and glia is unclear, as the currently identified co-factors may have a ubiquitous distribution [128,132,133,134,135].

## 7. Conclusions

Prion strain diversity is operationally defined by differences in tropism within and between tissues, with the regional distribution of PrP^Sc^ in the CNS being especially important (Figure 1). While the precise mechanism of prion tropism is unknown, the axonal transport of prions to clinical target areas may direct prions to populations of neurons and glia that contribute to disease pathology. Once there, the transition of a replicative form of PrP^Sc^ to that of a lethal form of PrP^Sc^ may trigger pathology. Additionally, strain-specific cellular cofactors may add further nuance to prion strain tropism.

## Data Availability

Not applicable.
